# Treatment of *Trypanosoma cruzi* with 2-bromopalmitate alters morphology, endocytosis, differentiation and infectivity

**DOI:** 10.1186/s12860-018-0170-3

**Published:** 2018-08-31

**Authors:** Cassiano Martin Batista, Rafael Luis Kessler, Iriane Eger, Maurilio José Soares

**Affiliations:** 1 Laboratory of Cell Biology, Carlos Chagas Institute/Fiocruz-PR, 81310-020 Curitiba, Paraná, Brazil; 2 Laboratory of Functional Genomics, Carlos Chagas Institute/Fiocruz-PR, 81310-020 Curitiba, Paraná, Brazil; 3 Mammalian Cell Biotechnology Laboratory, Molecular Biology Institute of Paraná (IBMP), 81310-020 Curitiba, Paraná, Brazil; 4 Department of General Biology, State University of Ponta Grossa, 84010-290 Ponta Grossa, Paraná, Brazil

**Keywords:** 2-Bromopalmitate, 2-BP inhibition, Differentiation, Endocytosis, Palmitoylation, *Trypanosoma cruzi*

## Abstract

**Background:**

The palmitate analogue 2-bromopalmitate (2-BP) is a non-selective membrane tethered cysteine alkylator of many membrane-associated enzymes that in the last years emerged as a general inhibitor of protein S-palmitoylation. Palmitoylation is a post-translational protein modification that adds palmitic acid to a cysteine residue through a thioester linkage, promoting membrane localization, protein stability, regulation of enzymatic activity, and the epigenetic regulation of gene expression. Little is known on such important process in the pathogenic protozoan *Trypanosoma cruzi*, the etiological agent of Chagas disease.

**Results:**

The effect of 2-BP was analyzed on different developmental forms of *Trypanosoma cruzi*. The IC_50_/48 h value for culture epimastigotes was estimated as 130 μM. The IC_50_/24 h value for metacyclic trypomastigotes was 216 nM, while for intracellular amastigotes it was 242 μM and for cell derived trypomasigotes was 262 μM (IC_50_/24 h). Our data showed that 2-BP altered *T. cruzi*: 1) morphology, as assessed by bright field, scanning and transmission electron microscopy; 2) mitochondrial membrane potential, as shown by flow cytometry after incubation with rhodamine-123; 3) endocytosis, as seen after incubation with transferrin or albumin and analysis by flow cytometry/fluorescence microscopy; 4) in vitro metacyclogenesis; and 5) infectivity, as shown by host cell infection assays. On the other hand, lipid stress by incubation with palmitate did not alter epimastigote growth, metacyclic trypomastigotes viability or trypomastigote infectivity.

**Conclusion:**

Our results indicate that 2-BP inhibits key cellular processes of *T. cruzi* that may be regulated by palmitoylation of vital proteins and suggest a metacyclic trypomastigote unique target dependency during the parasite development.

**Electronic supplementary material:**

The online version of this article (10.1186/s12860-018-0170-3) contains supplementary material, which is available to authorized users.

## Background

Palmitoylation is a post-translational protein modification that consists in addition of palmitic acid to a cysteine residue through a thioester linkage. This modification promotes membrane localization, regulation of enzymatic activity, regulation of gene expression and protein stability [1–3]. Palmitoylation is a reversible, dynamic modification regulated by enzymes that either transfer palmitic acid to a target protein (palmitoyl acyltransferases: PATs) or cleave the thioester linkages between palmitic acid and the modified proteins (palmitoyl protein thioesterases: PPTs) [4]. Palmitoylation represents an important modification in cells of a variety of organisms, such as mammals [5–7], yeasts [8, 9], fishes [10], plants [11, 12] and nematodes [13]. This modification is well characterized in humans, as it is strongly involved in Huntington’s disease and other neuropsychiatric diseases [14, 15] and cancer [16]. Palmitoylation was also recorded in pathogenic protozoa, including *Toxoplasma gondii* [17], *Plasmodium falciparum* [18] and *Trypanosoma brucei* [19].

The palmitate analogue 2-bromopalmitate (2-BP) is a non-selective membrane tethered cysteine alkylator of many membrane-associated enzymes that in the last years emerged as a general inhibitor of protein S-palmitoylation [20]. There are two proposed mechanisms for the 2-BP action: direct inhibition of PATs or blockage of palmitic acid incorporation by direct covalent competition with palmitate [21]. It has been suggested that 2-BP also inhibits PPTs, disturbing the acylation cycle of the protein GAP-43 at the depalmitoylation level and consequently affecting its kinetics of membrane association [22]. Incubation of the apicomplexan *T. gondii* with 50 μM 2-BP efficiently altered parasite morphology, gliding and host cell invasion [23]. In the African trypanosome *T. brucei*, the calculated IC_50_ values were 197 μM for the procyclic form and 226 μM for the bloodstream life form [19]. However, no 2-BP or global palmitoylation studies have been reported yet for *Trypanosoma cruzi*, a protozoan parasite that causes Chagas disease in Latin America.

Two *T. cruzi* proteins are known to be palmitoylated: TcFCaBP [24], which is involved in parasite motility, and TcPI-PLC [25], which is involved in evading the host immune system. A putative PAT has been identified in this protozoan (TcHIP/TcPAT1), localized in the Golgi complex of different life stages [26] and other nine could be overexpressed in epimastigotes, being mostly located at the anterior end of the parasites [27]. However, other still unidentified proteins should be also palmitoylated in *T. cruzi*, and palmitoylation is probably involved in diverse biological functions. A recent review on protein acylation in trypanosomatids with a focus on myristoylation and palmitoylation suggested that protein acylation represents an interesting target for the development of new trypanocidal drugs [28]. Indeed, *T. cruzi* N-myristoyltransferase (TcNMT), an enzyme that catalyzes the attachment of myristic acid to an N-terminal glycine residue of proteins, has been validated as a potential chemotherapeutic target in *T. cruzi* mammal stages [29].

The aim of this study was to assess the in vitro effect of 2-BP on *T. cruzi*. The data presented here show that this inhibitor alters the parasite morphology, endocytosis, differentiation and infectivity and suggest the importance of palmitoylation for parasite survival and its involvement in crucial biological processes.

## Methods

### Reagents

Trypan Blue, Dulbecco’s Modified Eagle Medium (DMEM), penicillin (10,000 units), streptomycin (10 mg/mL), trypsin from porcine pancreas, 2-bromopalmitate (2-BP), palmitate, dimethyl sulfoxide (DMSO), potassium chloride, propidium iodide, RNase A, carbonyl cyanide 3-chlorophenylhydrazone (CCCP), acridine orange, Giemsa stain, formaldehyde, paraformaldehyde, glutaraldehyde, Hoechst staining solution and poly-L-lysine were purchased from Sigma-Aldrich (St. Louis, MO, USA). Transferrin-AlexaFluor 633, Albumin-AlexaFluor 488, rhodamine-123, goat anti-mouse IgG AlexaFluor 488 conjugate, goat anti-mouse IgG AlexaFluor 594 conjugate and Prolong Gold were purchased from Molecular Probes/Life Technologies (Eugene, OR, USA). Potassium ferrocyanide, Permount, sodium cacodylate, osmium tetroxide and PolyBed-812 resin were purchased from Electron Microscopy Sciences (Hatfield, PA, USA). Sodium dodecyl sulfate (SDS) was purchased from Ludwig Biotecnologia (Alvorada, RS, Brazil). Fetal bovine serum (FBS) was purchased from Gibco/Invitrogen/Life Technologies (Eugene, OR, USA). Nonidet 40 (NP-40) was purchased from Anresco Laboratories (San Francisco, CA, USA).

### Vero cells

Vero cells (ATCC CCL-81) isolated from the kidney of the African green monkey *Cercopithecus aethiops*, were purchased from ATCC® (Washington DC, USA). The cells were maintained at 37 °C in 75-cm^2^ cell culture flasks (Corning Incorporated, Corning, NY, USA) in DMEM medium supplemented with 10% FBS in a humidified 5% CO_2_ atmosphere. For weekly seeding, the cell monolayers were washed twice with PBS, trypsinized and the detached cells were collected by centrifugation for 5 min at 800×*g*. The cells were then inoculated at 10^6^ cells/flask in fresh DMEM medium and kept as described above.

### Trypanosoma cruzi

Culture epimastigote forms of *T. cruzi* clone Dm28c, isolated from *Didelphis marsupialis* in Venezuela [30] were maintained at 28 °C by weekly passages in Liver Infusion Tryptose (LIT) medium [31] supplemented with 10% heat-inactivated fetal bovine serum (FBS).

In vitro-derived metacyclic trypomastigotes were obtained by incubating epimastigotes in Triatomine Artificial Urine (TAU/TAU3AAG) medium, according to a previously described metacyclogenesis (i.e., epimastigote-to-trypomastigote differentiation) protocol [32], with a yield of approximately 50%. Metacyclic trypomastigotes were purified with a DEAE-cellulose column as previously described [32].

Cell-derived trypomastigotes were obtained from Vero cell cultures infected with in vitro-derived metacyclic trypomastigotes, at a ratio of 100 parasites/cell. After 4 h of interaction the host cell monolayers were washed with PBS to remove the non-adherent parasites. Infected cells were then incubated for six days in 10 mL of DMEM medium supplemented with 10% FBS, when trypomastigote production peaked. The culture supernatant was collected, and the cell-derived trypomastigotes released into the supernatant were harvested by centrifugation for 15 min at 3,000 *g*. The parasites were then used for the experiments and to maintain the infection cycle.

### Amplification of PATs genes

DNA sequences of fourteen TcPATs (PATs 2–15) were identified and used for primer design [27] with subsequent amplification by PCR, using genomic DNA (gDNA) of *T. cruzi* (Dm28c) epimastigotes. gDNA was extracted from three-day-old culture epimastigotes by a phenol-chloroform method [33]. TcPAT1 (TcHIP) primers [26] were also used for PCR. Amplifications were confirmed by 1.0% agarose gel electrophoresis.

### Determination of IC_50_ value for 2-BP

Stock solutions at 100 mM of 2-BP and palmitate were prepared in DMSO. The solutions were filtered through a 0.22-μm Millipore filter (Merck Millipore Co, Tullagreen, CO, Ireland) and stored at 4 °C. After dilution in culture medium, the DMSO concentration in the experiments never exceeded 1%, and it did not affect parasite growth.

To calculate the concentration of 2-BP that inhibited 50% growth of the epimastigote cultures (IC_50_/48 h), the parasites (10^6^/mL) were incubated at 28 °C with different concentrations of 2-BP (25 to 400 μM) in biological triplicates. Cell counts were made after 48 h with a Neubauer chamber. The population density was calculated, and the death percentage was estimated relative to the untreated control (LIT medium with 1% DMSO), generating dose-effect curves. The CompuSyn software [34] was then used to calculate the IC_50_/48 h value by using the death percentage for each 2-BP concentration. For morphological analysis, the parasites were processed for bright field, scanning and transmission electron microscopy as described below.

To calculate the IC_50_/24 h for metacyclic and cell-derived trypomastigotes, the parasites (10^6^ cells/mL) were incubated with different concentrations of 2-BP (0.1 to 175 μM for metacyclic trypomastigotes; 0.1 to 400 μM for cell-derived trypomastigotes) in biological triplicates. After 24 h at 28 °C (or 4 h at 37 °C for culture trypomastigotes, to avoid differentiation into extracellular amastigote-like forms) the parasite number was counted in a Neubauer chamber and the death percentage was calculated relative to the untreated control (culture medium with 1% DMSO). The CompuSyn software was used to calculate the IC_50_ value.

To calculate the IC_50_ value for intracellular amastigotes, 24-h-old infected Vero cell cultures (10^6^ cells/mL) were incubated with 2-BP (0.4 to 125 μM) in biological triplicates in a humidified 5% CO_2_ atmosphere. After 24 and 48 h of treatment, the infected host cells were lysed by nitrogen decompression [35] and the number of released amastigotes was counted in a Neubauer chamber. Population density was then compared to the untreated control (medium with 0.125% DMSO). The CompuSyn software was used to calculate the IC_50_ value. The number of released trypomastigotes was then evaluated after 72 h of treatment (96 h of infection).

The IC_50_/24 h for intracellular amastigotes was calculated with purified amastigotes obtained from 48-h-old infected cells cultures, by lysing the host cells with nitrogen decompression [35]. After three steps of centrifugation at low speed to remove the Vero cells, the purified intracellular amastigotes were incubated at 37 °C with DMEM medium in 6-well plates (approximately 10^7^ parasites/well) in a humidified 5% CO_2_ atmosphere. This cell suspension was centrifuged after 30 min to remove remaining intact Vero cells and new DMEM medium (containing additional 1000 units penicillin and 100 μg/mL streptomycin) was added. The purified isolated intracellular amastigotes were then incubated for 24 h with 2-BP (0.4 to 500 μM) in biological triplicates. The CompuSyn software was used to calculate the IC_50_ value.

The 2-BP IC_50_ values obtained from the experiments on epimastigote growth and metacyclic trypomastigote viability were used in control assays of lipidic stress, by incubating these forms (48 h at 28 °C for epimastigotes, 24 h at 28 °C for metacyclic trypomastigoes) with palmitate.

### Cytotoxicity to Vero cells

To calculate the cytotoxicity for Vero cells (CC_50_/24 h), 10^5^ cells/mL were cultivated in 6-well plates in biological triplicates. After 24 h, the cultures were washed twice with PBS and incubated at 37 °C with different concentrations of 2-BP (75 to 300 μM) in a humidified 5% CO_2_ atmosphere. After 24 h of treatment, cell cultures were washed twice with PBS, trypsinized, washed in DMEM medium, stained with Trypan blue (0.02% final concentration) for cell viability analysis and counted in Neubauer chamber. The percentage of dead cells was estimated relative to the untreated control (culture medium with 0.3% DMSO). The CompuSyn software was used to calculate the CC_50_/24 h value.

### Flow cytometry of *T. cruzi* epimastigotes

Flow cytometry experiments were performed with a FACS Aria-II (Becton-Dickinson, San Jose, CA, USA). A total of 20,000 events were acquired in the region previously identified as corresponding to *T. cruzi* epimastigotes [36]. The data were analyzed using the FlowJo software package (FlowJo, Ashland, OR, USA). Epimastigotes (2 × 10^6^ cells) were incubated for 48 h with the IC_50_ value of 2-BP (130 uM), washed twice with PBS and then used in the experiments.

For cell cycle assays, epimastigote DNA was stained with propidium iodide (PI). The parasites were incubated with cell cycle buffer (3.4 mM Tris-HCl, 30 μg/mL PI, 0.1% NP-40, 10 mM sodium chloride, 700 U/L RNAse, pH 7.6) and immediately analyzed using a 610/20 nm bandpass filter. Cell debris and doublets were excluded using a width × area gate. The Dean-Jett-Fox algorithm of FlowJo was used to estimate the percentage of cells in the G1, S and G2/M phases of the cell cycle.

For mitochondrial membrane potential analysis, epimastigotes were incubated with IC_50_/48 h 2-BP or palmitate and then incubated for 15 min at room temperature with 10 μg/mL rhodamine-123. After two washes with PBS, the parasites were analyzed using a 530/30 nm bandpass filter. CCCP at 100 μM for 5 min was used as a positive control of the mitochondrial membrane potential destabilization [37]. The relative mitochondrial membrane potential was determined by considering normalized median ratios (treated/control) of the fluorescence level. The normalized fluorescence medians were used for statistical analysis.

For cell viability assay, IC_50_/48 h 2-BP treated epimastigotes were incubated for 5 min at room temperature with PI (5 μg/mL) and then analyzed without washing. Positively stained cells (610/20 nm bandpass filter) were considered dead.

For acid compartment analysis, the IC_50_/48 h 2-BP treated epimastigotes were incubated for 15 min at room temperature with 5 μg/mL acridine orange, washed twice with PBS and then analyzed using a 695/20 nm bandpass filter. The normalized fluorescence medians were analyzed as described above [36]. In parallel, parasites were adhered to glass slides and visualized with epifluorescence microscopy using a B-2A (long pass emission) filter (Nikon, Chiyoda, Japan).

### Endocytosis assays

Epimastigotes were incubated for 4 h with IC_50_/48 h 2-BP, washed twice in PBS and subjected to a previously described endocytosis assay [35, 38] using 2 × 10^6^ parasites for flow cytometry analysis or 5 × 10^6^ cells for fluorescence microscopy studies. After 15 min under stress in PBS at 25 °C, the parasites were incubated for 30 min at 28 °C with 50 μg/ml transferrin-AlexaFluor 633 or albumin-AlexaFluor 488. Negative control cells were incubated in the absence of labeled transferrin or albumin. For flow cytometry, living epimastigotes were analyzed using 660/20 and 530/30 nm bandpass filters for transferrin-AlexaFluor 633 or albumin-AlexaFluor 488 acquisition, respectively. The normalized median fluorescence intensity of transferrin and albumin was calculated as the ratio between the median fluorescence intensity of the treated and untreated cells. Data acquisition and analysis was performed as described above [35].

### 2-BP treatment during in vitro metacyclogenesis

Mid log-phase epimastigotes were collected and submitted to various treatments with IC_50_/48 h 2-BP during metacyclogenesis: (a) Control: five days in LIT/0.13% DMSO and then incubation for 2 h in TAU and 72 h in TAU3AAG media without 2-BP; (b) Pre-stress: five days in LIT medium with IC_50_/48 h 2-BP followed by incubation for 2 h in TAU without 2-BP; (c) Stress: five days in LIT medium and then 2 h in TAU medium containing with IC_50_/48 h 2-BP; and (d) Post-stress: five days in LIT medium, stress for 2 h in TAU medium and incubation for 15 min in TAU3AAG with IC_50_/48 h 2-BP.

After 2-BP treatments the cells were washed twice with PBS to remove 2-BP and incubated for 72 h in TAU3AAG medium. The parasites in the supernatants were then collected and counted in Neubauer chamber to obtain the ratio of metacyclic trypomastigotes / epimastigotes per mL. For morphological analysis, the parasites were processed for light microscopy.

### Infection assays

Metacyclic trypomastigotes obtained from 2-BP treatments during metacyclogenesis (see above) were used to infect Vero cells. Briefly, 10^7^ metacyclic trypomastigotes were incubated with 10^6^ Vero cells and after 24 h noninvasive trypomastigotes were removed by PBS washes. After 6 days, the infected cultures were washed with PBS and the released trypomastigotes were collected from the PBS by centrifugation. The trypomastigotes were then counted and processed for light and fluorescence microscopy analysis.

In other experiment, cell-derived trypomastigotes were treated with IC_50_/4 h 2-BP or palmitate and then used to infect Vero cells. Briefly, the parasites (2 × 10^7^ cells/mL) were pre-incubated with IC_50_/4 h 2-BP or palmitate, washed twice with PBS, counted in Neubauer chamber and then incubated for 2 h with 10^6^ adhered Vero cells. Non-adherent parasites were then washed out with PBS. Four days after infection the released trypomastigotes were collected, counted in Neubauer chamber and processed for light and fluorescence microscopy. Vero cell cultures infected with untreated trypomastigotes were used as a control.

### Light microscopy

The parasites were adhered for 10 min to 0.1% poly-L-lysine coated slides, fixed for 2 min at room temperature with methanol, air dried and then incubated for 30 min at room temperature with Giemsa stain. The samples were then washed with MilliQ water and mounted with Permount. After 24 h the slides were observed with a Nikon E600 microscope (100× objective) in bright field mode for image acquisition.

### Fluorescence microscopy

To detect the native TcFCaBP, epimastigotes and metacyclic trypomastigotes were incubated with the respective IC_50_ values of 2-BP or palmitate, washed twice in PBS, fixed for 20 min with 4% formaldehyde and incubated for 1 h at room temperature with incubation buffer (1.5% BSA/PBS). This step was followed by incubation for 1 h at 37 °C with a primary mAb 25 monoclonal antibody against TcFCaBP [39], which was diluted to 1:1000 in incubation buffer. The samples were then washed three times in PBS and incubated for 1 h at 37 °C with a secondary goat anti-mouse antibody coupled to AlexaFluor-488 or AlexaFluor-594 (1:600) in incubation buffer.

To co-localize transferrin-AlexaFluor-633 or albumin-AlexaFluor-488 with cruzipain (CZP), the epimastigotes subjected to the endocytosis assays were fixed for 30 min in 4% paraformaldehyde and permeabilized for 5 min with 0.5% Triton in PBS. For co-localization of CZP/transferrin-AlexaFluor-633, the samples incubated with CZP-315.D9 mAb (at 50 μg/ml) [40], were washed three times in PBS and then incubated with a secondary goat anti-mouse-AlexaFluor-488 antibody (1:600 in incubation buffer). For the co-localization with CZP/albumin-AlexaFluor-488, the samples were incubated with CZP-315.D9 mAb and then incubated with a secondary goat anti-mouse-AlexaFluor-594 antibody.

After antibody incubations, all samples were washed three times in PBS, incubated for 5 min with 1.3 nM Hoechst 33342, washed twice in PBS and twice in MilliQ Water, mounted with Prolong Gold anti-fading reagent and then examined under a Nikon Eclipse E600 epifluorescence microscope.

### Scanning electron microscopy

Parasites were washed twice in PBS and fixed for 1 h at room temperature in 2.5% glutaraldehyde in 0.1 M phosphate buffer, pH 7.2. The fixed parasites were washed twice in 0.1 M cacodylate buffer and then adhered for 15 min at room temperature to 0.1% poly-L-lysine coated coverslips. The non-adhered cells were washed out twice with 0.1 M cacodylate buffer and the adhered cells were then incubated for 15 min at room temperature with 1% osmium tetroxide diluted in 0.1 M cacodylate buffer. The samples were washed three times with 0.1 M cacodylate buffer and dehydrated in a graded acetone series (30%, 50%, 70%, 90% and 100% acetone, 5 min each). This step was followed by critical point drying in a Leica EM CPD300 and gold sputtering in a Leica EM ACE200. The samples were visualized in a JEOL JSM-6010 PLUS/LA scanning electron microscope at 20 kV.

### Transmission electron microscopy

Parasites were washed twice in PBS and fixed for 1 h in 2.5% glutaraldehyde in 0.1 M phosphate buffer at room temperature. The fixed parasites were washed twice in 0.1 M cacodylate buffer and then post-fixed with 1% osmium tetroxide / 1.6% potassium ferrocyanide / 5 mM CaCl_2_ diluted in 0.1 M cacodylate buffer for 30 min at room temperature. The samples were washed three times with 0.1 M cacodylate buffer, dehydrated in a graded acetone series (5 min in 30, 50, 70, 90 and 100%) and then embedded in PolyBed812 resin. Ultrathin sections were obtained in a Leica EM UC6 ultramicrotome, collected on copper grids, contrasted with uranyl acetate and lead citrate and then visualized in a JEOL 1400Plus transmission electron microscope at 90 kV.

### Statistical analysis

All experiments were performed in independent biological triplicates. Statistical analysis was performed using the software GraphPad Instat (GraphPad Software Inc., La Jolla, CA, USA) for ANOVA followed by a Bonferroni’s multiple comparisons test.

## Results

### PATs genes in *T. cruzi*

PCR using specific primers for each gene encoding TcPATs resulted in amplification of fourteen genes with the expected number of base pairs (Additional file 1: Figure S1), except for TcPAT6 (no positive amplification).

### Effect of 2-BP on different *T. cruzi* developmental forms

**Fig. 1:**
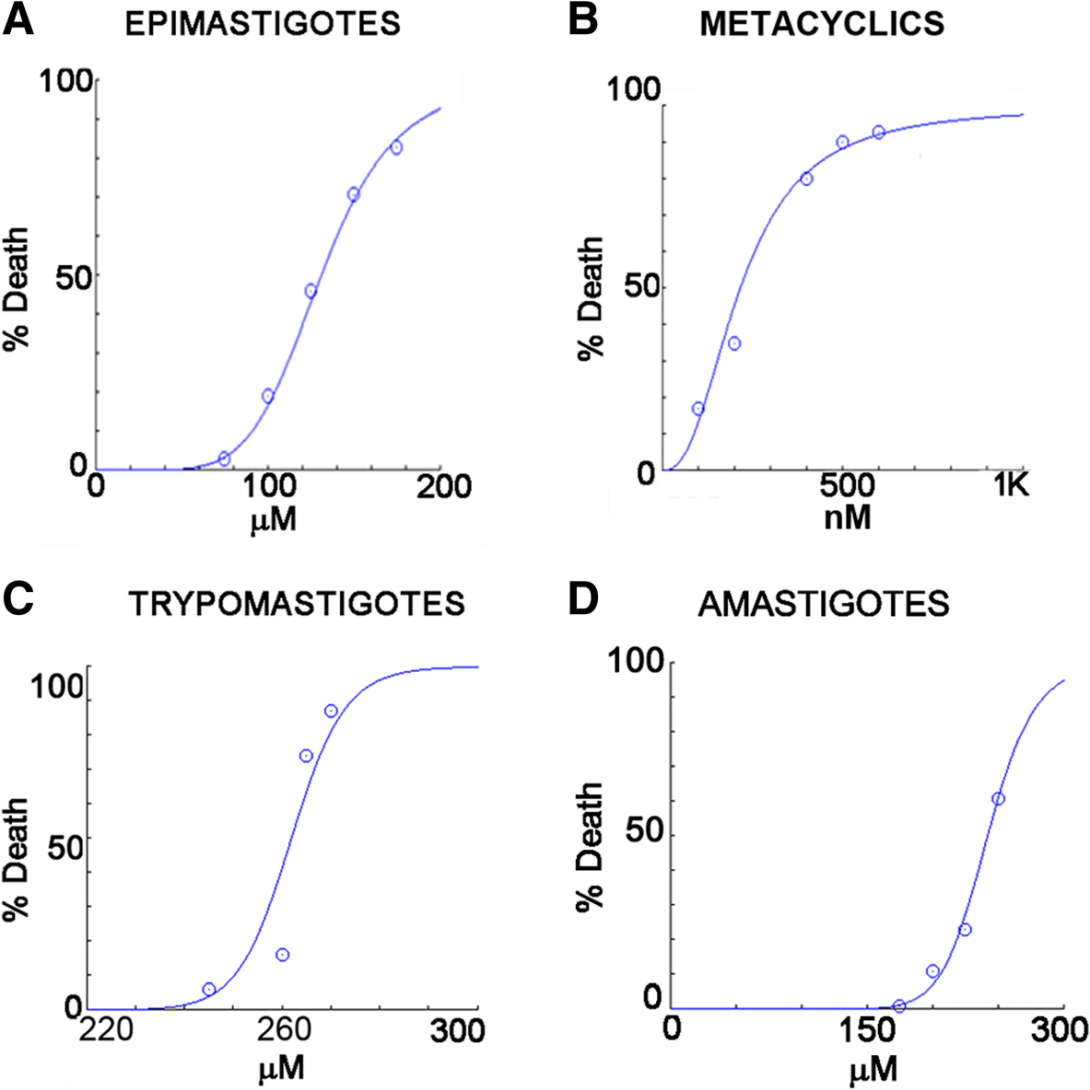
Effect of 2-BP on different *Trypanosoma cruzi* developmental forms. **a** Effect on epimastigotes. IC_50_ value was estimated as 130 μM. *n* = 3, *p* < 0.001. **b** Effect on metacyclic trypomastigotes. IC_50_/24 h value was estimated as 216 nM. *n* = 3, *p* < 0.001. **c** Effect on culture trypomastigotes. IC_50_/4 h value was estimated as 262 μM. *n =* 3, *p* < 0.001. **d** Effect on isolated intracellular amastigotes. IC_50_ = 242 μM. *n =* 3, *p* < 0.001

The IC_50_/48 h value of 2-BP for culture epimastigotes was estimated as 130 μM (Fig. 1a). The estimated IC_50_ value (IC_50_/24 h) of 2-BP for metacyclic trypomastigotes was 216 nM (Fig. 1b), while for cell-derived trypomastigotes it was 262 μM (Fig. 1c).

The cytotoxic effect (CC_50_/24 h) of 2-BP on Vero cells was calculated before the assays with intracellular amastigotes, and its estimated value was 138 μM. Concentrations higher than 200 μM killed 100% of the host cells. No effect on number and morphology of intracellular amastigotes was observed after 24 or 48 h of treatment of infected Vero cells with up to 125 μM 2-BP (data not shown). However, 125 μM 2-BP enhanced the intracellular differentiation into trypomastigote forms.

Isolation of intracellular forms by nitrogen decompression after 24 h of treatment showed several trypomastigote-like stages, characterized by the presence of a bar kinetoplast close to the nucleus (Additional file 2: Figure S2). There was an increase of approximately 40% in the number of released trypomastigotes after four days of infection (72 h of treatment) when compared to untreated cultures.

Finally, intracellular amastigotes were isolated from the host cells by nitrogen decompression and were then incubated with different concentrations of 2-BP. In this case, the estimated IC_50_/24 h value was 242 μM (Fig. 1d). This high concentration could explain why we failed to calculate the IC_50_ value for intracellular amastigotes (no effect up to 125 μM).

### Mislocalization of TcFCaBP after incubation with 2-BP

**Fig. 2:**
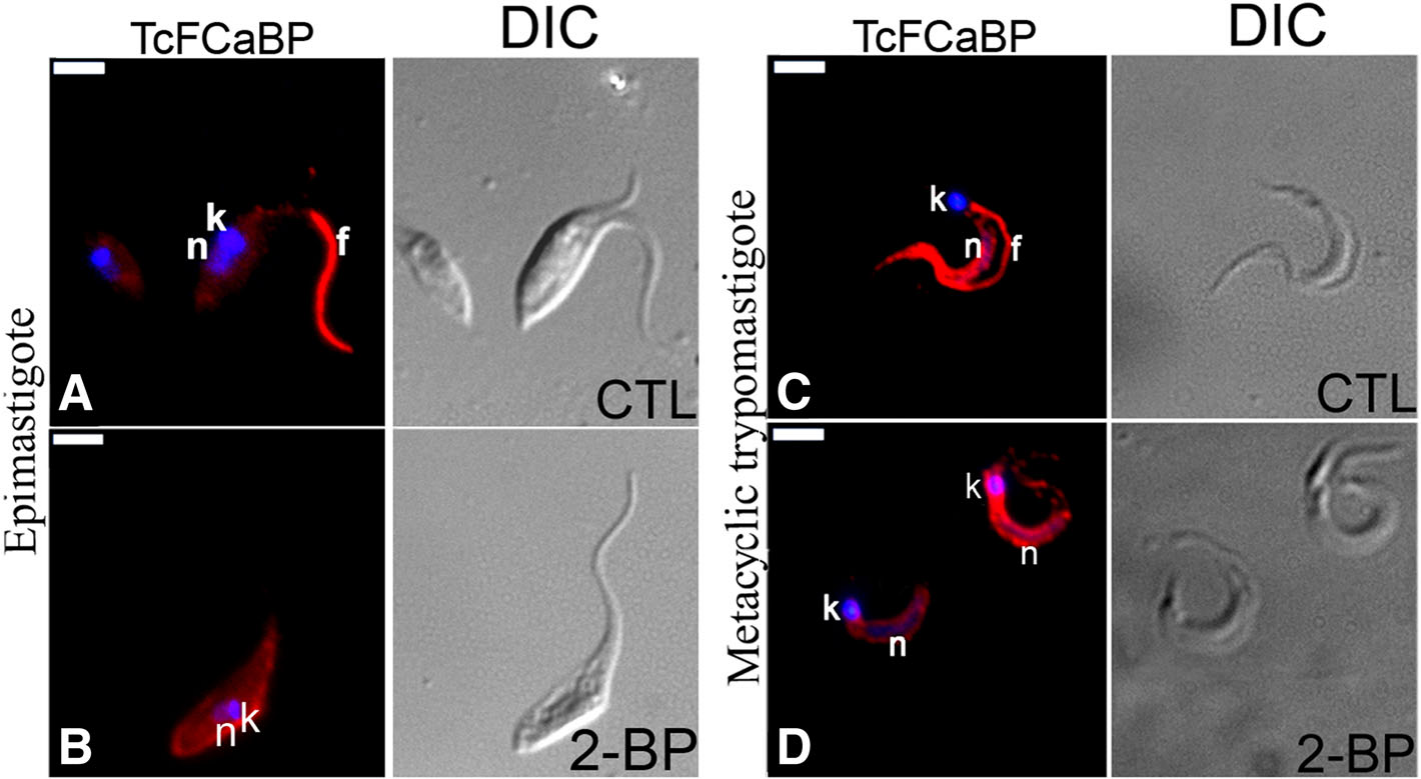
Localization of TcFCaBP in Trypanosoma cruzi epimastigotes and metacyclic trypomastigotes after 2-BP treatment. **a**-**c** Negative control (CTL), showing flagellar localization (in red) of TcFCaBP in epimastigotes (**a**) and trypomastigotes (**c**). Nucleus and kinetoplast are stained with DAPI (blue). **b**-**d** Incubation with 130 μM 2-BP hindered flagellar localization of TcFCaBP. n: nucleus; k: kinetoplast; f: flagellum. Bars = 5 μm

Epimastigotes and metacyclic trypomastigotes were treated respectively for 48 or 24 h with their 2-BP IC_50_ values and then incubated with a monoclonal antibody against TcFCaBP, a flagellar calcium-binding protein known to be palmitoylated [24, 38]. While untreated epimastigotes (Fig. 2a) and metacyclic trypomastigotes (Fig. 2c), showed prominent flagellar labeling, the 2-BP-treated cells lost their flagellar labeling and showed a disperse reaction throughout the cell body (Fig. 2b and d).

### Morphology, viability and physiology of *T. cruzi* epimastigotes were altered by treatment with IC_50_/48 h 2-BP

**Fig. 3:**
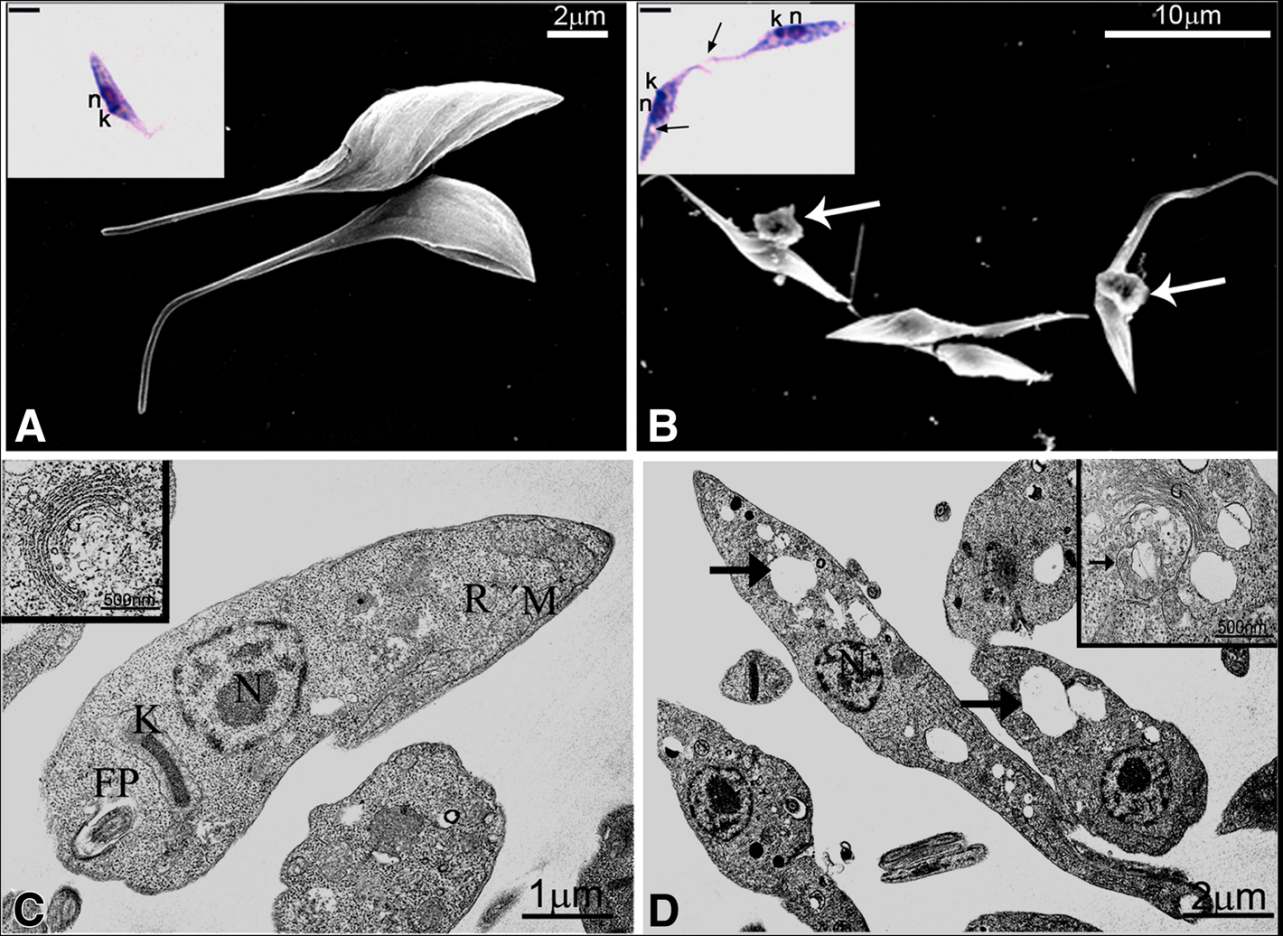
Morphological alterations of *Trypanosoma cruzi* epimastigotes treated with IC_50_/48 h 2-BP. **a**-**b** Scanning electron microscopy of control (**a**) and 2-BP-treated (**b**) parasites. Note the extracellular leakage at the flagellar pocket region of treated epimastigotes (write arrows). Insets (bar = 5 μm): bright field microscopy of Giemsa-stained parasites. Control parasite (in **a**) showing the characteristic elongated shape; treated parasites (in **b**) were larger, with large vacuoles and were adhered by their flagella (black arrows). **c**-**d** Transmission electron microscopy. Control epimastigote (**c**) showing the typical elongated morphology. A representative Golgi complex is shown in the inset. 2-BP-treated parasites (**d**) presented large electron lucent vacuoles at the anterior tip (black arrows) and Golgi complex alterations (arrow in inset). N: nucleus; K: kinetoplast; FP: flagellar pocket; G: Golgi complex; R: reservosome; M: mitochondrion. Bars = 5 μm

2-BP treated epimastigotes had translucent vacuoles at the posterior region and were occasionally linked by the flagella (insets in Fig. 3a and b). Scanning electron microscopy showed a leakage of intracellular material at the flagellar pocket region (Fig 3a and b). Transmission electron microscopy showed large electron lucent vacuoles at the posterior cell region and Golgi alterations (Fig. 3c and d).

To certify that the large vacuoles observed by light and transmission electron microscopy were reservosomes (acidic pre-lysosomal organelles found at the posterior end of *T. cruzi* epimastigotes), 2-BP-treated epimastigotes were incubated with acridine orange. Analysis by flow cytometry showed a 2-fold increase in the median red fluorescence intensity peak (Additional file 3: Figure S3, left panel). Using fluorescence microscopy, a strong red labeling was found in structures at the posterior region of the treated cells (Additional file 3: Figure S3, right panel), thus indicating that these large vacuoles corresponded to reservosomes.

**Fig. 4:**
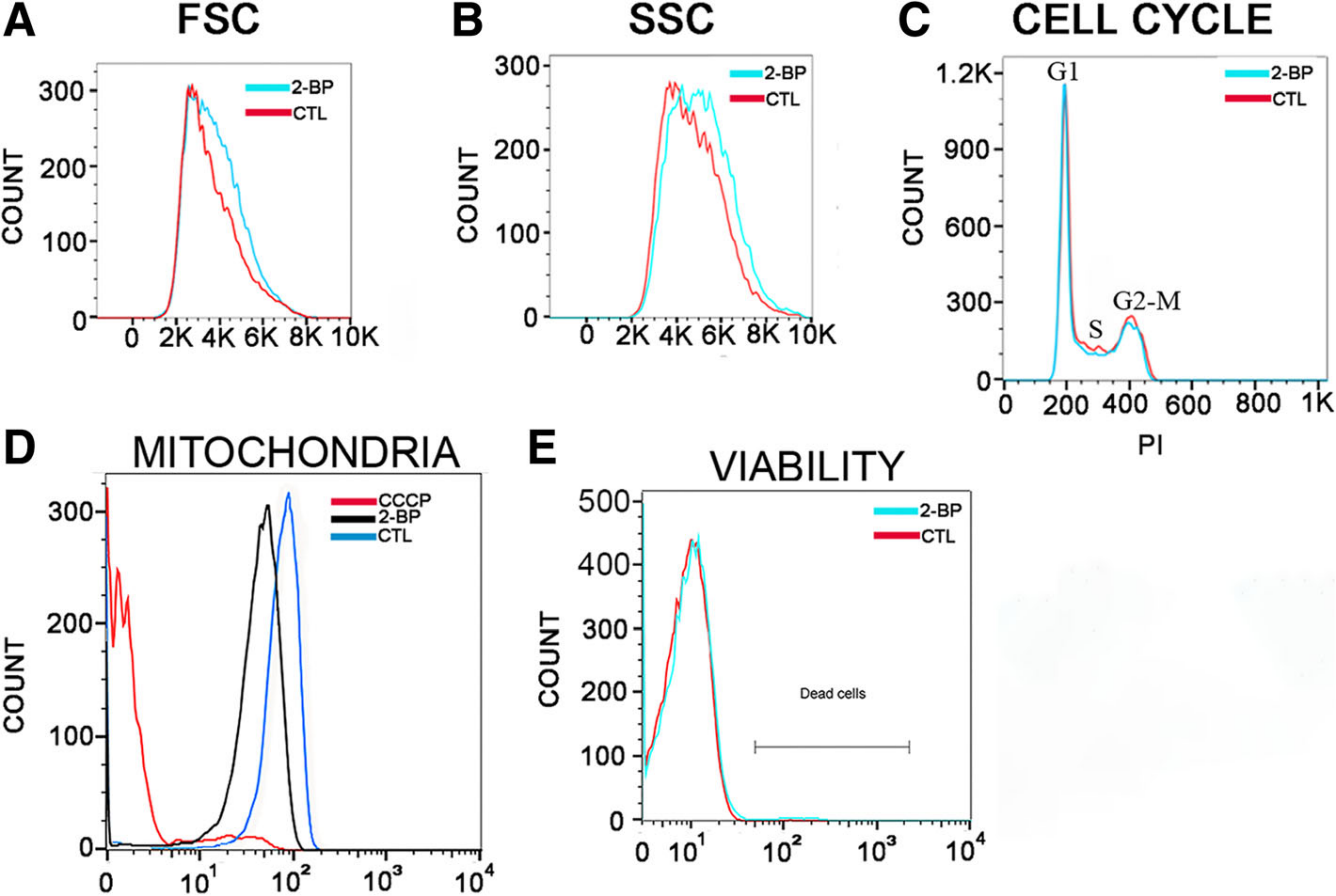
Effect of incubation with IC50/48 h 2-BP on Trypanosoma cruzi epimastigotes as assessed by flow cytometry. **a** Forward scatter (FSC) analysis of control (CTL) and treated parasites, showing the larger size of the treated epimastigotes. **b** Side scatter (SSC) analysis of CTL and 2-BP parasites, showing an increase in granularity. **c** Cell cycle analysis, showing no difference between CTL and 2-BP treated parasites in the different cell cycle stages. **d** Mitochondrial potential analysis by rhodamine-123, showing a decrease in the membrane potential for 2-BP-treated epimastigotes; 100 μM CCCP: positive control. **e** Cell viability analysis of CTL and 2-BP parasites by propidium iodide, showing a few dead cells in 2-BP-treated cultures

The size (FSC) and granularity (SSC) of 2-BP-treated epimastigotes were analyzed by flow cytometry, and they showed statistically significant increases of approximately 13% and 11% (*p* ≤ 0.0012 and *p* ≤ 0.007), respectively, compared to the control (Fig 4a and b). There was no significant difference in the distribution of cells in the different cell division cycle stages (G1, S and G2-M) between the treated and the control parasites (Fig. 4c).

In mitochondrial viability assays with rhodamine-123, the normalized median of the fluorescence peaks in the stained cells decreased 47% (*p* ≤ 0.001) in the 2-BP-treated epimastigotes (Fig. 4d), showing that the mitochondrial potential was altered by the palmitoylation inhibition. Only 1.5% of the population was dead after 48 h of incubation with 2-BP (Fig. 4e), thus indicating that most parasites were viable.

### Endocytosis in *T. cruzi* epimastigotes is hindered by 2-BP

Epimastigotes were treated for 4 h with 130 μM 2-BP, washed and then incubated for 30 min in LIT medium containing endocytic markers. Two different tracers were used: transferrin (mostly internalized via the cytostome) and albumin [41].

**Fig. 5:**
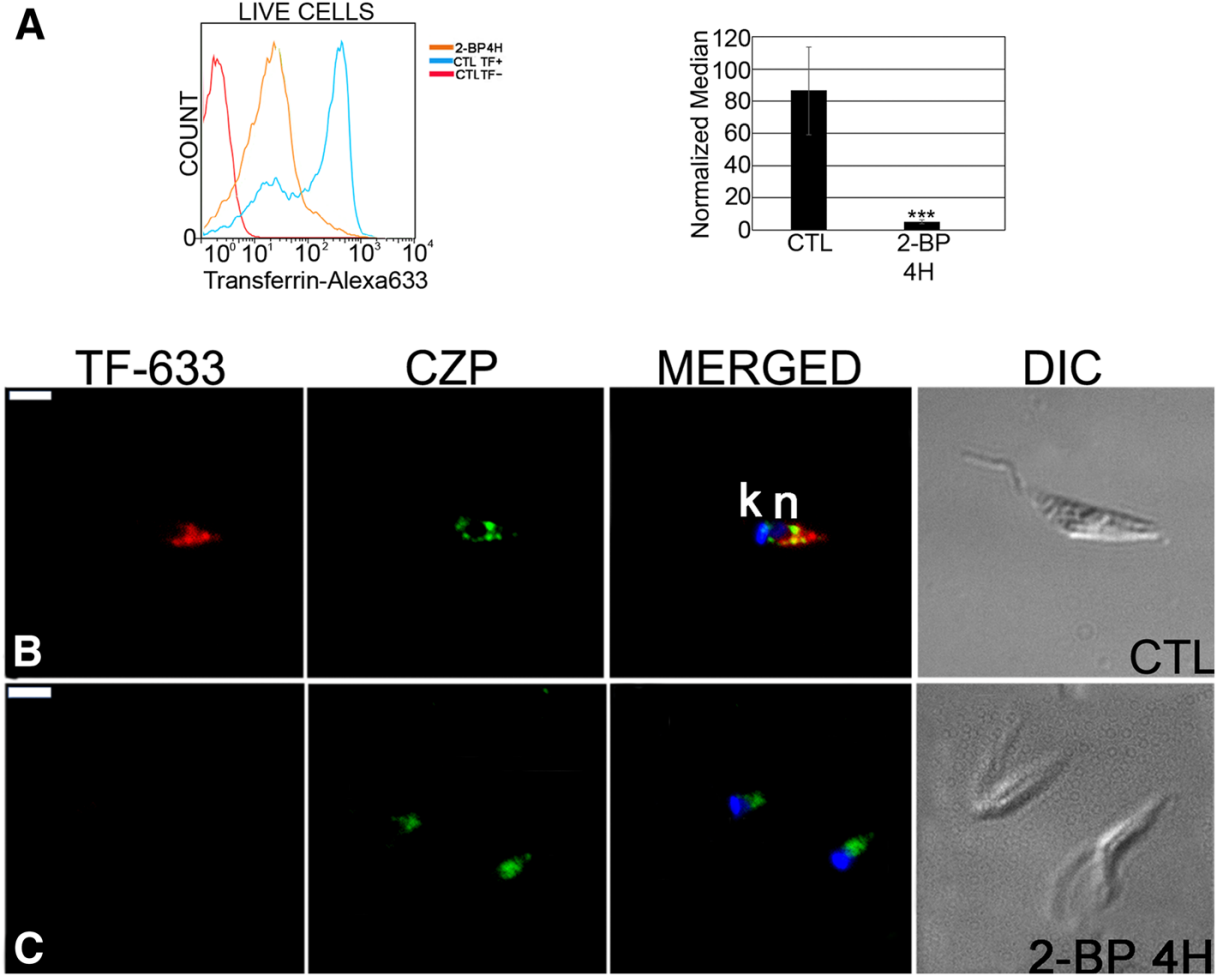
Transferrin-AlexaFluor 633 endocytosis is altered in *Trypanosoma cruzi* epimastigotes by 2-BP treatment. **a** Flow cytometry analysis showing that transferrin internalization was inhibited after 4 h of treatment with 2-BP. ***: *p* < 0.001. **b** Control cell: co-localization of internalized transferrin (TF-633) with cruzipain (CZP) in reservosomes by fluorescence microscopy. **c** Parasites treated for 4 h with 2-BP showing no fluorescence signal for transferrin. n: nucleus; k: kinetoplast. Bars = 5 μm

Incubation with transferrin-Alexa 633 and analysis by flow cytometry showed that 2-BP treated parasites had low endocytic activity, with an approximately 90% reduction in the normalized 633 fluorescence median (Fig. 5a). Fluorescence microscopy showed that untreated cells internalized transferrin partially in co-localization with the reservosomal marker cruzipain (Fig. 5b). On the other hand, transferrin fluorescence was reduced in 2-BP parasites and no co-localization with cruzipain was observed (Fig. 5c).

**Fig. 6:**
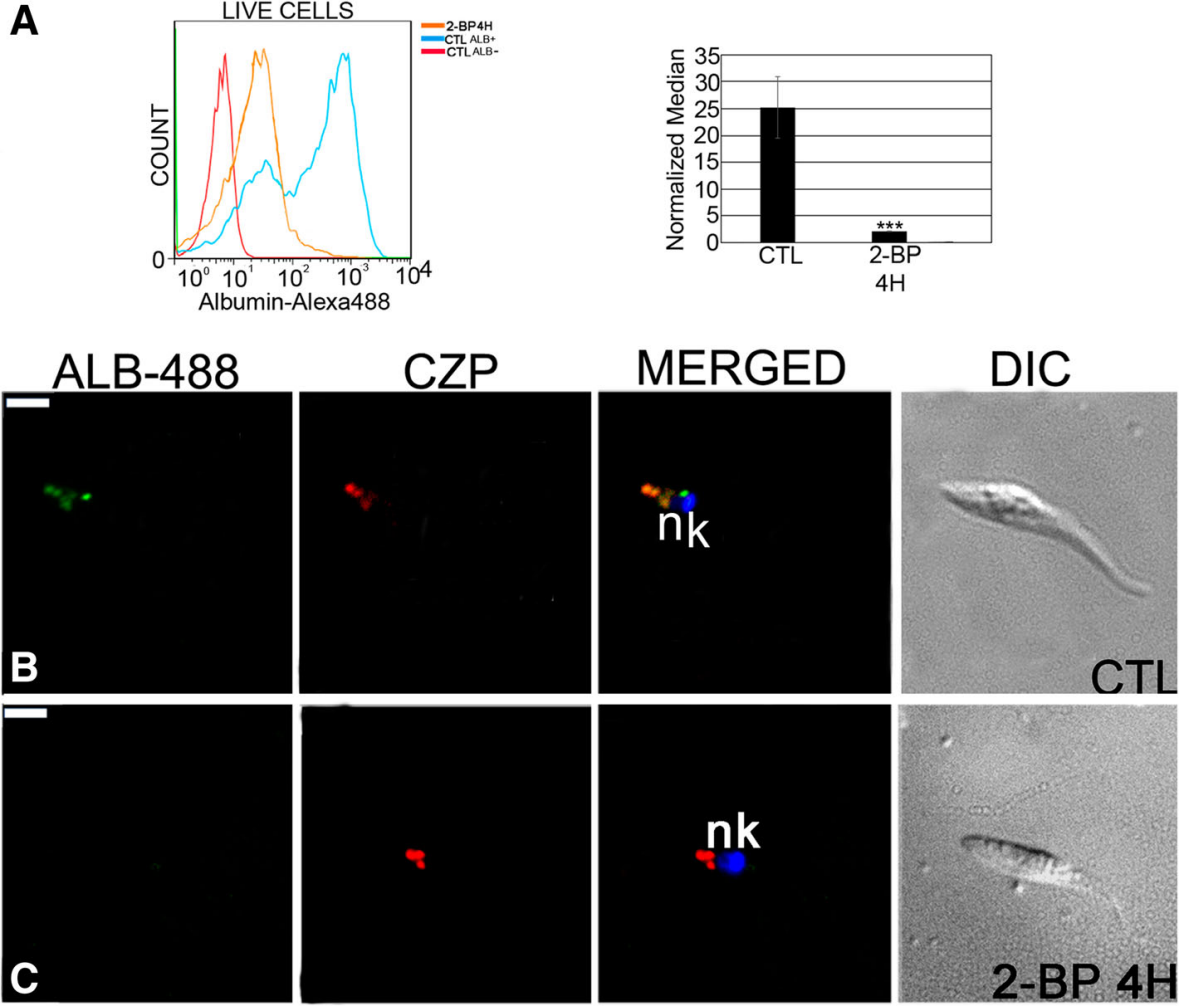
Albumin-Alexa 488 endocytosis is altered in *Trypanosoma cruzi* epimastigotes by 2-BP treatment. **a** Flow cytometry analysis showing that albumin internalization was inhibited after treatment for 4 h with 2-BP. ***: *p* < 0.001. **b** Control: Co-localization of internalized albumin (ALB-488) with cruzipain (CZP) in reservosomes by fluorescence microscopy. **c** Parasites treated for 4 h with 2-BP showing no fluorescence signal of albumin (no co-localization with cruzipain). N: nucleus; k: kinetoplast. Bars = 5 μm

Treated epimastigotes incubated with albumin-Alexa 488 showed an approximately 90% reduction in normalized 488 fluorescence (Fig. 6a). Fluorescence microscopy showed no co-localization with cruzipain and little albumin was ingested (Fig. 6b and d).

### *Trypanosoma cruzi* metacyclogenesis is altered by 2-BP

**Fig. 7:**
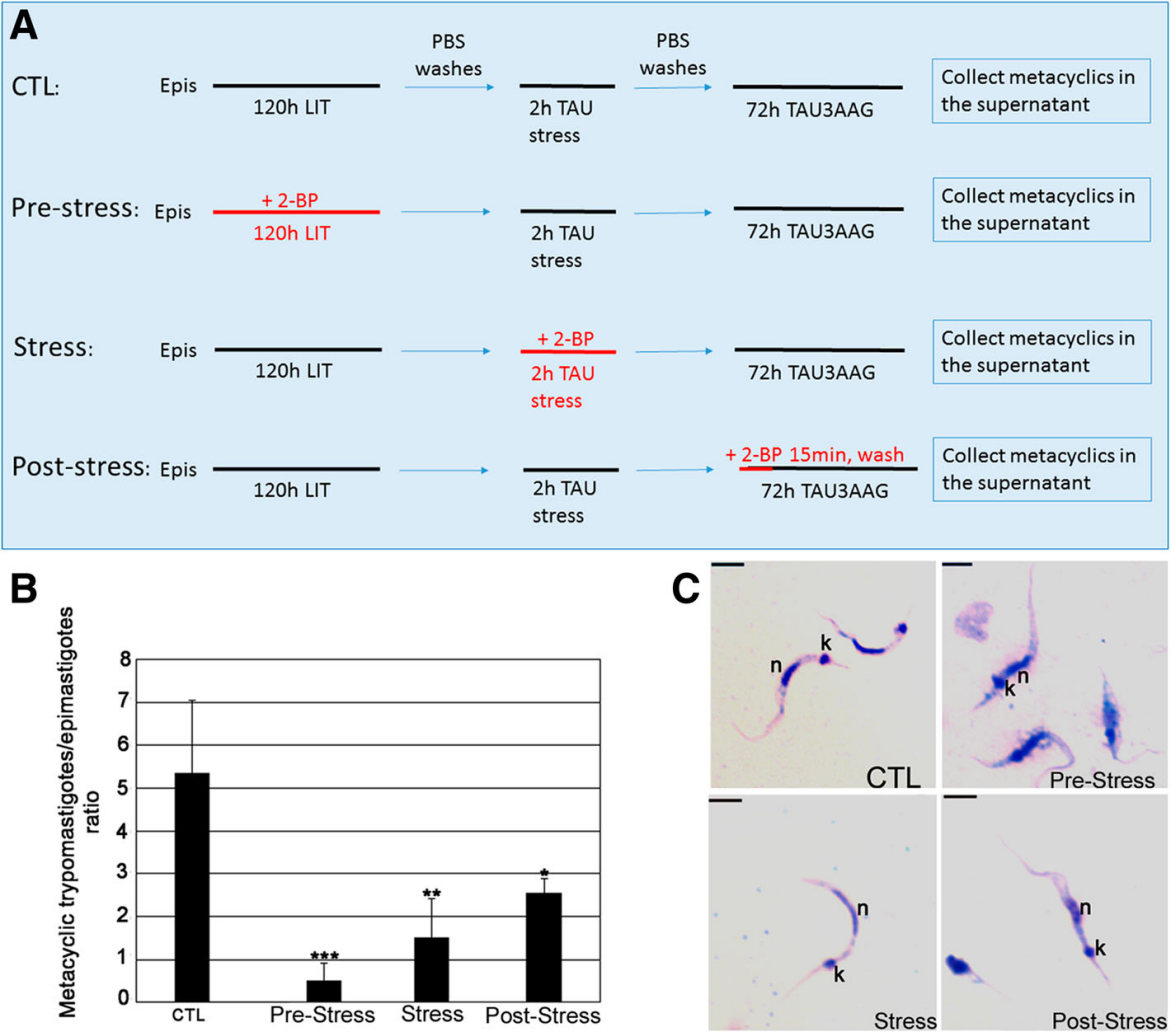
Metacyclogenesis of *Trypanosoma cruzi* is inhibited by 2-BP treatment. **a** Schematic view of the metacyclogenesis experimental design. **b** Ratio of metacyclic trypomastigotes/epimastigotes after 72 h in the supernatant of TAU3AAG medium; the ratio decreased in all treatments, when compared to the untreated control. *: *p* > 0.005; **: *p* < 0.005; ***: *p* < 0.001. **c** Giemsa-stained cells collected in the supernatant showing morphological alterations after the different treatments. n: nucleus; k: kinetoplast. Bars = 5 μm

The metacyclic trypomastigotes/epimastigotes ratio was evaluated after 72 h in the supernatant of the TAU3AAG differentiation medium (Fig. 7a). While in the control this ratio was approximately 5:1, for 2-BP-treated parasites the ratio ranged from 0.5:1 (2-BP in pre-stress medium) to 2.5:1 (2-BP in post-stress medium) (Fig. 7b). Incubation with TcFCaBP mAb showed that all treatments (2-BP in pre-stress, stress or post-stress media) led to mislocalization of this flagellar protein, which was found at the parasite surface, but not at the flagellum.

Morphology of parasites from the culture supernatants was analyzed by light microscopy. Metacyclic trypomastigotes were abundant in the untreated control (Fig. 7c). When 2-BP was added to pre-stress assays, then epimastigotes prevailed (Fig. 7c) and the few detected metacyclic trypomastigotes were smaller, with their nucleus and kinetoplast closely located. When 2-BP was added to the stress and post-stress assays, round cells prevailed and the few detected metacyclic trypomastigotes were morphologically similar to those of the control (Fig. 7c).

### Infectivity of *T. cruzi* trypomastigotes is altered by 2-BP

**Fig. 8:**
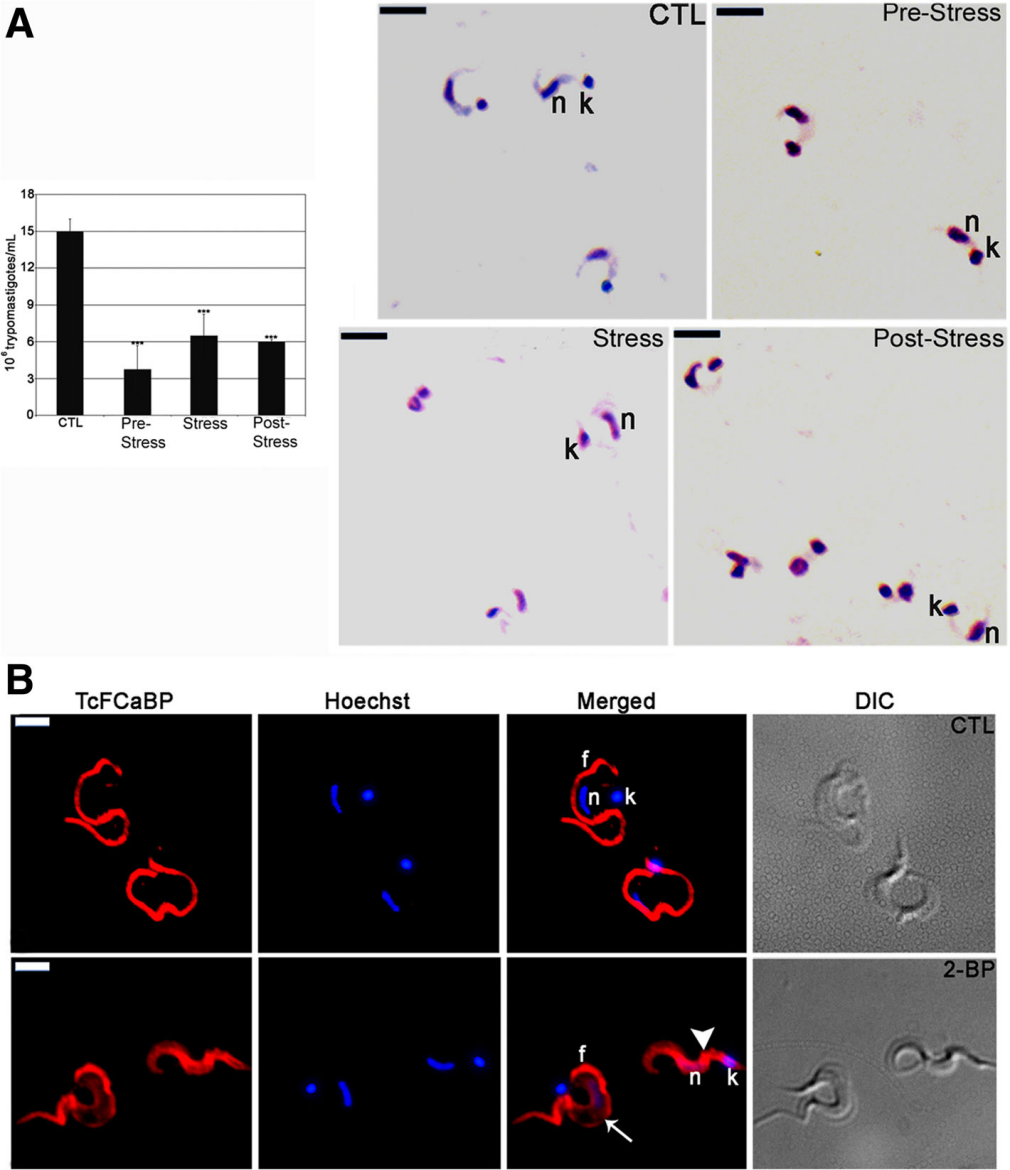
2-BP treatment during metacyclogenesis alters *Trypanosoma cruzi* host cell infectivity. **a** Number of released cell-culture trypomastigotes (CTL and 2-BP-treated groups) after Vero cell infection, showing a reduction of approximately 45.5% to 75% for the treated groups when compared to the control parasites. *n =* 3, ***: *p* < 0.001. Released cell-culture trypomastigotes (CTL and 2-BP-treated groups) as visualized by light microscopy showing morphological alterations, such as smaller size and round nucleus, compared to the control parasites. n: nucleus; k: kinetoplast. Bars = 5 μm. **b** Localization of TcFCaBP of *Trypanosoma cruzi* trypomastigotes released from Vero cells infected with 2-BP-treated metacyclic trypomastigotes (pre-stress group). CTL: Negative control showing a strong labeling in the flagellum. 2-BP: Treated parasites showing cellular (arrowhead) and partial flagellar (arrow) labeling. n: nucleus; k: kinetoplast; f: flagellum. Bars = 5 μm

Trypomastigotes obtained from the TAU3AAG supernatants of the 2-BP metacyclogenesis assay were used to infect Vero cells. Six days after infection, the released trypomastigotes were counted and the parasite number was compared to that of trypomastigotes collected from Vero cell cultures that were infected with untreated parasites. There was a significant inhibition in the number of released trypomastigotes for all 2-BP treatments, ranging from 48.5 to 75% (Fig. 8a).

Light microscopy showed round-shaped, smaller and thicker parasites with a round nucleus in all treated groups when compared to the control trypomastigotes (Fig. 8a). Released trypomastigotes from the control and pre-stress assays were incubated with TcFCaBP mAb and were visualized by fluorescence microscopy (Fig. 8b). In the control parasites protein localization was enriched in their flagella (Fig. 8b), while in the trypomastigotes from the 2-BP experiments the localization was diffuse on the cell surface or was partially located at the flagellar membrane (Fig. 8b).

**Fig. 9:**
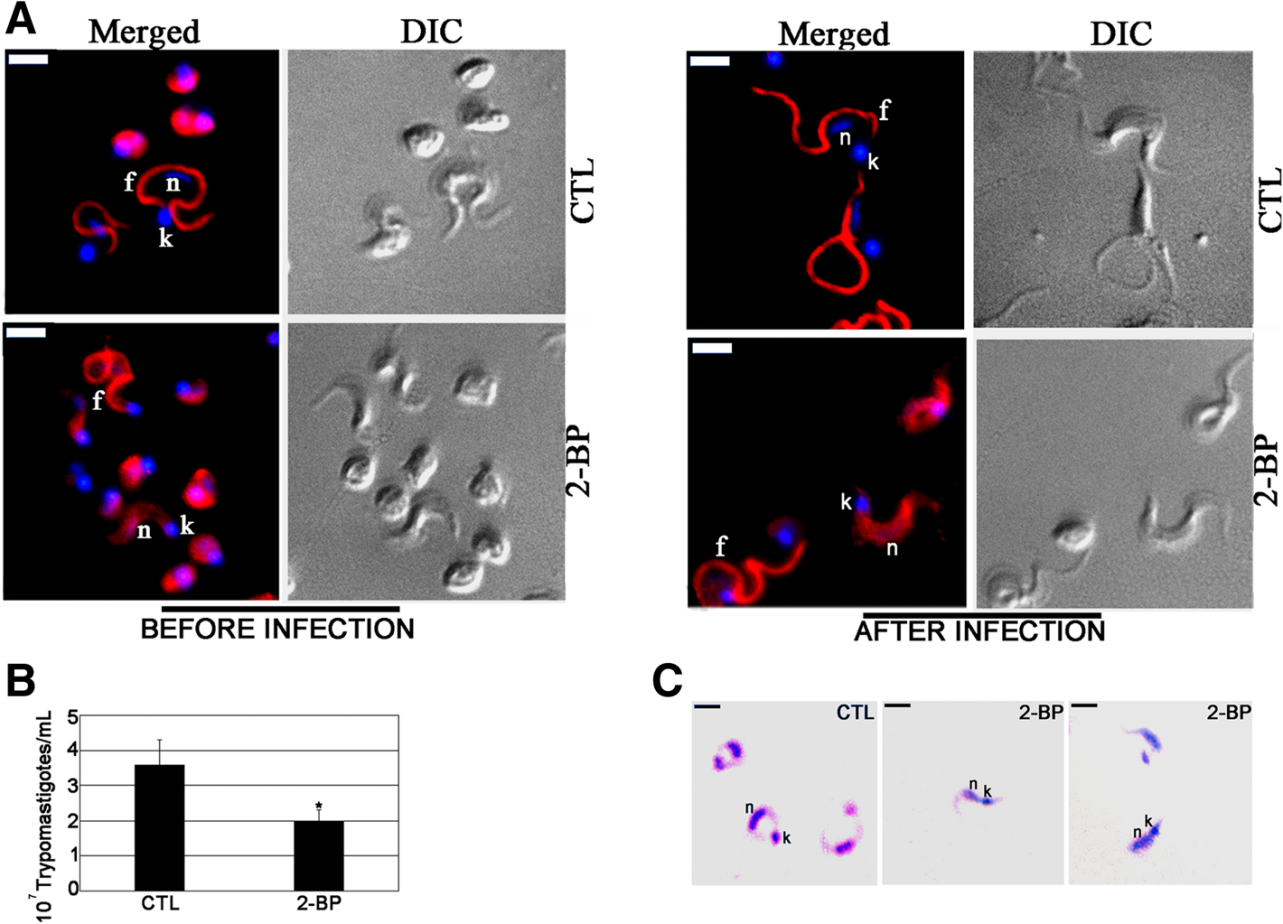
2-BP treatment of culture trypomastigotes with IC_50_ 2-BP alters *Trypanosoma cruzi* host cell infectivity. **a** 2-BP treatment before infection altered TcFCaBP flagellar localization in parasites obtained before and after infection. **b** Note the decrease in number of released trypomastigotes four days after infection. *: *p* > 0.05 (**c**) Giemsa staining of released trypomastigotes showing morphological alterations. n: nucleus; k: kinetoplast. Bars = 5 μm

In another experiment, cell-derived trypomastigotes were treated with IC_50_ 2-BP (262 μM) and then used to infect Vero cells. At this experimental point (before infection), aliquots of the treated and untreated (control) trypomastigotes were incubated with the TcFCaBP mAb. In untreated trypomastigotes the labeling was in the flagellum, while in treated parasites the labeling was diffuse in the cytoplasm, with a few trypomastigotes presenting flagellar labeling (Fig. 9a, “before infection”). After four days of infection the released trypomastigotes were collected and incubated with the TcFCaBP mAb. The same result was obtained: while in trypomastigotes from the control experiment the labeling was in the flagellum (Fig. 9a, “after infection”), in trypomastigotes obtained from the 2-BP treatment assay the labeling was diffuse in the cytoplasm, with a few trypomastigotes presenting flagellar labeling (Fig. 9a, “after infection”). The released trypomastigotes were counted and their numbers were compared. There was a 45.5% inhibition in the number of released parasites in the treatment assay (Fig. 9b). The morphology of the released parasites from the treatment assay was analyzed by light microscopy, showing round-shaped, smaller, thicker cells with a rounded nucleus, when compared to the control trypomastigotes (Fig. 9c).

### Effect of palmitate on different *T. cruzi* developmental forms

**Fig. 10:**
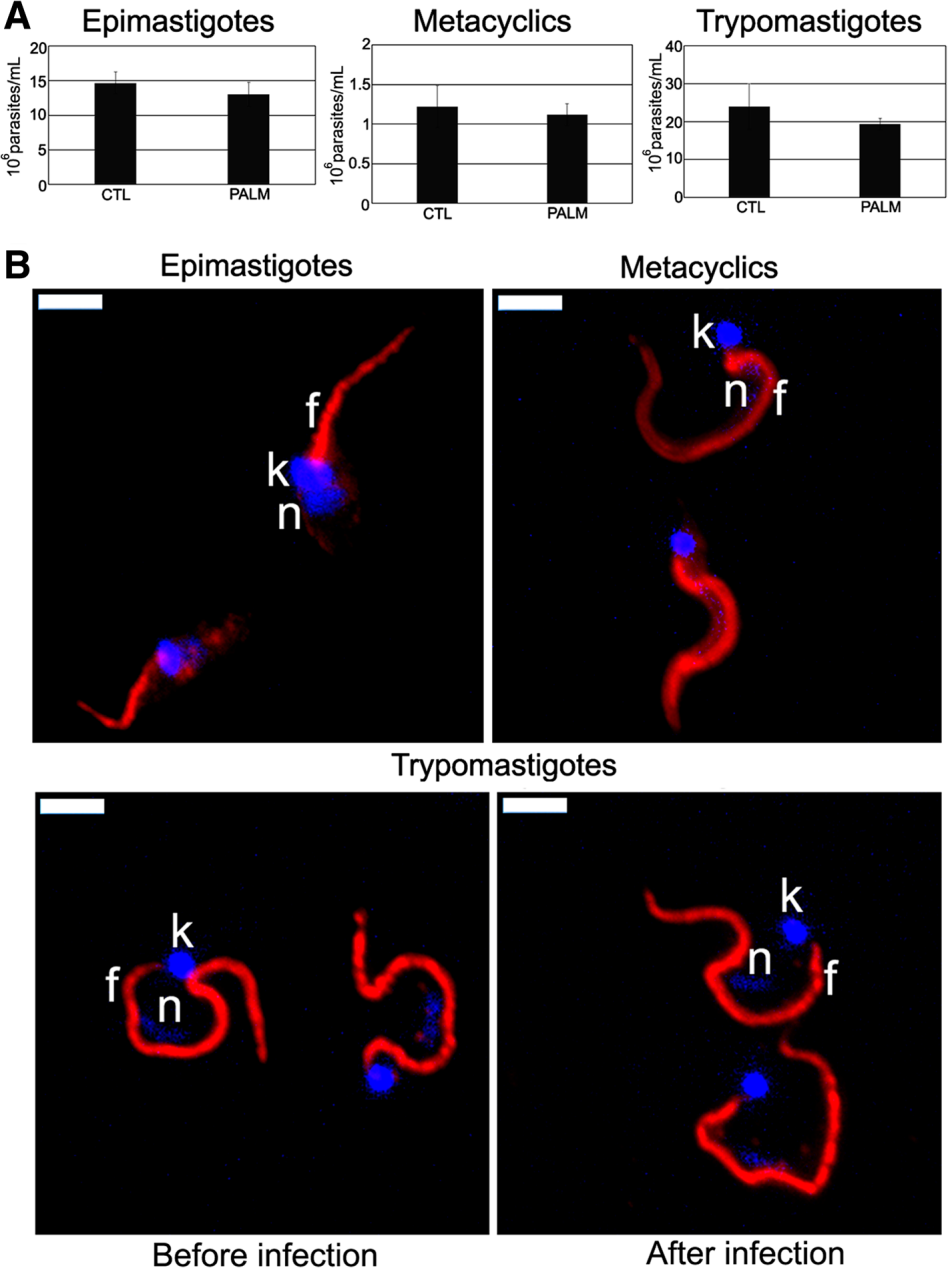
Effect of palmitate on *Trypanosoma cruzi*. Different developmental forms were incubated with palmitate, using the respective IC_50_ values of 2-BP. **a** No effect was observed on epimastigotes growth, metacyclics viability and cell-derived trypomastigotes infectivity. **b** Localization of TcFCaBP was not altered in all developmental forms tested. n: nucleus; k: kinetoplast; f: flagellum. Bars = 5 μm

Epimastigotes treated with 130 μM (IC_50_/48 h value) palmitate showed no significant alteration in growth, the parasite number decreasing only in 13.4% when compared to untreated cells (Fig. 10a, epimastigotes). Metacyclic trypomastigotes remained alive after incubation with 216 nM (IC_50_/24 h value), the parasite number decreasing only in 8.5% when compared to untreated cells (Fig. 10a, metacyclics). Treatment of cell-derived trypomastigotes for 4 h with 262 μM (IC_50_/4 h value) palmitate had low effect on infectivity, with decrease of 19.5% in the number of released trypomastigotes (Fig. 10a, trypomastigotes). Furthermore, TcFCaBP subcellular localization was not altered in all these developmental forms (Fig. 10b).

Epimastigotes treated with 130 μM (IC_50_/48 h value) palmitate showed a reduction in 27.5% of the mitochondrial potential after rhodamine 123 incubation (Additional file 4: Figure S4), as compared to 47% reduction in 2-BP treated cells.

## Discussion

Protein palmitoylation promotes membrane localization, regulation of enzymatic activity, regulation of gene expression and protein stability [1–3]. Many palmitoylated proteins are important for diverse aspects of pathogenesis in eukaryotic parasites, including differentiation into infective life cycle stages, biogenesis and tethering of secretory organelles, assembling the machinery powering motility and targeting virulence factors to the plasma membrane [42]. Here we analyzed the effect 2-BP, a palmitate analogue that can inhibit palmitoylation, on the different life stages of the pathogenic protozoan *T. cruzi*.

*T. cruzi* has two main developmental forms when in the vertebrate host: intracellular amastigotes and bloodstream trypomastigotes. Assays on amastigotes are relevant as amastigotes are responsible for tissue damage and trypomastigote formation [43]. The existence of an intracellular epimastigote-like form as an intermediate stage within the mammalian host, morphologically and biochemically similar to the non-infectious extracellular epimastigote form [44], supports the preliminary screening of compounds on the non-infectious stage of the parasite [45]. Therefore, data concerning all *T. cruzi* developmental forms (epimastigotes, amastigotes and trypomastigotes) are crucial to explore the basic cell biology of *Trypanosoma cruzi*.

The repertoire of *T. cruzi* palmitoyl transferases (PATs) was first checked and 15 putative proteins were found [27], which agrees with Goldston and coworkers [28]. All respective genes, except for TcPAT6, were successfully isolated by PCR, thus indicating that *T. cruzi* has the palmitoylation machinery in its genome. Indeed, dynamic S-palmitoylation machinery could be expressed in *T. cruzi* epimastigotes [27]. Therefore, 2-BP could be exploited to gain some information on this lipid modification in this parasite, since it has been already demonstrated that 2-BP inhibits some PATs in *T. brucei* [19].

*T. cruzi* culture epimastigotes incubated with 130 μM 2-BP (IC_50_/48 h) did not show flagellar localization of TcFCaBP (a flagellar protein that is known to be palmitoylated), which agrees with the data obtained with *T. cruzi* TcFCaBP palmitoylation-deficient mutants (C4A and ΔN) [24], thus indicating that 2-BP also inhibits palmitoylation in *T. cruzi* epimastigotes. TcFCaBP was also mislocated in metacyclic and culture trypomastigotes that were incubated with an IC_50_ value of 2-BP (216 nM and 262 μM, respectively), thus suggesting that palmitoylation could be important modification for protein localization in *T. cruzi*. While the data points to a new pharmacological sensitivity to 2-BP in the metacyclic stage, this could just as likely be a dependence on fatty acid synthesis, or any number of other cellular enzymes that are inhibited by micromolar concentrations of 2-BP, as it has been shown that 2-BP is broadly reactive across hundreds of cellular proteins at low micromolar concentrations in mammalian cells [21]. On the other hand, trypanosomatids are unicellular organism with a large evolutionary distance to mammalian cells [46, 47]. The earliest forms of *T. cruzi* itself are deduced to have been associated with marsupial opossums at the time of separation of South America from Gondwanaland about 40 million years ago [48]. Consequently, it is also possible that 2-BP is not so broadly reactive in *T. cruzi* due to the parasite own protein repertoire. Further studies are needed to clarify this point.

Interestingly, incubation with palmitate did not alter epimastigote growth, metacyclic trypomastigote viability and TcFCaBP flagellar localization in the different developmental forms, thus indicating that the alterations observed in our study were due to the 2-BP treatment. However, it is still unclear if 2-BP operates by targeting a specific mechanism, as it has been shown that 2-BP is a promiscuous inhibitor of membrane-bound enzymes [21, 49]. Further experiments are needed to elucidate this point.

Incubation of infected Vero cells for 72 h with 2-BP resulted in a 40% increase in the number of released trypomastigotes. It is possible that 2-BP treatment led to deregulation of metabolic pathways (e.g., energy production, nucleotide metabolism, pteridine biosynthesis and/or fatty acid oxidation) in the host cells or in the intracellular parasites, which are key processes for the parasite intracellular development [50], accelerating the parasite intracellular differentiation cycle. Indeed, several enzymes and transporters for the above mentioned metabolic pathways were already identified in the *T. brucei* palmitoylome [19].

2-BP-treated epimastigotes showed marked morphological alterations. The large vesicles close to the Golgi complex that were observed by transmission electron microscopy could be a result of inhibition of palmitoylation, leading to accumulation of depalmitoylated proteins in vesicles at the trans-Golgi network, since it is known that the Golgi compartment acts as a hub for palmitoylation [51]. Moreover, mostly overexpressed FLAG-tagged TcPATs were found as single spots at the parasite anterior end, which could be the Golgi complex [27]. It is possible that the vesicles observed close to the Golgi could leave the flagellar pocket by exocytosis, thus forming the extracellular material observed by scanning electron microscopy.

2-BP-treated epimastigotes showed a reduction of 47% in mitochondrial membrane potential, as opposed to 27.5% in palmitate-treated parasites, thus indicating that the membrane potential alteration was more likely due to the 2-BP effect than to a lipidic stress. It has been recently shown that an active and dynamic S-depalmitoylation is present in mitochondria, regulating S-palmitoylation [52]. It is thus tempting to speculate that S-palmitoylation also occurs in the *T. cruzi* mitochondrion.

Internalization of transferrin and albumin was inhibited in the 2-BP-treated epimastigotes. Our data on transferrin inhibition agree with a former work on the inhibition of diferric transferrin receptor-mediated endocytosis that is associated with palmitoylation of the transferrin receptor [53], thus suggesting that the transferrin receptor of *T. cruzi* could be palmitoylated. However, the existence of a transferrin receptor in *T. cruzi* has been proposed [54], but this receptor has been not yet identified. Our data indicate that the two endocytic portals of *T. cruzi* epimastigotes, the cytostome and the flagellar pocket [38, 55], are deficient for transferrin/albumin internalization when the parasites were treated. Accordingly, the pellet of 2-BP-treated epimastigotes was paler than that of the control parasites (data not shown), indicating that treated parasites were deficient in incorporating hemin from the LIT medium.

The translucent vacuoles observed by light microscopy at the posterior end of epimastigotes corresponded to the large, electron-lucent vacuoles found by transmission electron microscopy. Incubation with acridine orange showed an approximately 99% increase of the fluorescence signal by flow cytometry and a stronger red labeling by fluorescence microscopy at the posterior cell end, indicating that these large vacuoles correspond to the reservosomes, acidic organelles that accumulate ingested proteins [55, 56]. The lower endocytic capacity of 2-BP-treated epimastigotes resulted in the appearance of these less dense reservosomes.

It has been proposed that the content of reservosomes is metabolized during the metacyclogenesis process [56]. Considering that the 2-BP-treated epimastigotes had poor endocytic activity, we analyzed the effect of incubating epimastigotes with 2-BP before metacyclogenesis (pre-stress assay). As a result, treated epimastigotes had a decreased ability to differentiate (up to 75%). Light microscopy showed that some resulting metacyclic forms had a nucleus close to the kinetoplast, indicating that the development of the differentiation process was affected. Therefore, it seems that epimastigotes bearing reservosomes with low protein content have decreased aptitude for the differentiation process.

All morphological alterations found in 2-BP stressed epimastigotes indicate that the treatment was highly detrimental for differentiation and infectivity. When the resulting metacyclic trypomastigote forms were submitted to an infection assay, the percentage of released trypomastigotes decreased from 48.5 to 25%, demonstrating that the treatment interfered with parasite infectivity, possibly due to loss of surface proteins involved in host cell interactions. Metacyclic trypomastigotes obtained from the pre-stress metacyclogenesis assays were used to infect Vero cell cultures. As a result, released trypomastigotes (i.e., after 10 days without treatment with 2-BP) still showed TcFCaBP mislocalization and nuclear/kinetoplast morphological alterations, which could contribute to reduce the number of released parasites.

We submitted cell-derived trypomastigotes treated with IC_50_ 2-BP to a host cell interaction assay to determine whether the treatment could result in reduced infectivity. The number of released trypomastigotes decreased 45.5% in the treated group, demonstrating that the treatment interfered with host cell interactions. Compared to the control trypomastigotes, the treated parasites had round-shaped, smaller, thicker cell bodies with a round nucleus, together with mislocalization of TcFCaBP. On the other hand, low reduction in infectivity and no TcFCaBP mislocalization were found in palmitate treated parasites. These results suggest that palmitoylation was altered in 2-BP treated trypomastigotes.

Our data indicate that 2-BP affects the morphology, endocytosis, differentiation and infectivity of *T. cruzi*, thus suggesting that these functions could be somehow linked to protein palmitoylation. Our major finding on metacyclic trypomastigotes suggests a unique target dependency during *T. cruzi* development that is suitable for pharmacological rationales. The next step is to identify and validate the biochemical pathways involved with palmitoylation that lead to these alterations by proteomic and reverse genetic approaches. Future studies focusing on the characterization of these pathways are also paramount to understand the role of palmitoylation-dependent protein localization in parasite survival.

Click-enabled activity-based probes have been used for profiling the targets of 2-BP inhibition. It has been shown that the probes preferentially labeled the active site of DHHC PATs, but similarly labeled hundreds of other proteins, including transporters, channels, enzymes, and chaperones [21]. Therefore, it is recognized that 2-BP is more likely a non-selective membrane tethered cysteine alkylator with many targets beyond palmitoyl transferases [21, 49]. Thus, although data on the sensitivity of *T. cruzi* for 2-BP cannot be linked solely to palmitoylation, we cannot rule out the possibility that palmitoylation is involved in some or all events here reported (due to the TcFCaBP mislocalization). The challenging search of specific palmitoylation inhibitors is a crucial step to determine the role of palmitoylation on the *T. cruzi* biology.

Some palmitoylation inhibitor compounds have been already described [57], and one of them - Compound V (CV) - behaved similarly to 2-BP, in that it inhibited all four of the DHHC proteins tested. 2-BP and CV inhibited autoacylation of the PAT enzyme, which is tightly correlated with the ability to transfer palmitate to substrate [58]. Future works with other specific palmitoylation inhibitors against *T. cruzi* could elucidate the role played by palmitoylation in the parasite life cycle and could lead to a potential inhibitor of parasite infection with validated targets.

## Conclusions

2-bromopalmitate treatment of *Trypanosoma cruzi* altered parasite morphology, endocytosis, differentiation and infectivity**,** indicating that 2-BP inhibits key cellular processes of *T. cruzi* that may be regulated by palmitoylation of vital proteins. Palmitoylation is an important cellular process that may be a good target for further cellular/molecular biology studies with specific palmitoylation inhibitors in order to elucidate the life cycle of *T. cruzi*. Our major finding on metacyclic trypomastigotes suggests a unique target dependency during *T. cruzi* development that is suitable for pharmacological rationales.

## Additional files

Additional file 1: **Figure S1.** *Trypanosoma cruzi* PATs genes amplification by PCR as analyzed by 1% agarose gel. Note the expected amplifications for all PATs genes, except TcPAT6. Kb = 1 Kb plus ladder. (TIF 141 kb)

Additional file 2: **Figure S2.** 48 h-old *Trypanosoma cruzi* intracellular parasites isolated by cavitation after 24 h with 125 μM 2-BP. A) Control isolated amastigote incubated in DMEM medium with 0.125% DMSO; B) Treated parasite with an intermediate trypomastigote-like morphology, with kinetoplast close to the nucleus; C) Treated parasite showing the typical trypomastigote form. *n =* nucleus; k = kinetoplast. Bars = 5 μm. (TIF 332 kb)

Additional file 3: **Figure S3.** Characterization of acid compartments in control (CTL) and 2-BP-treated epimastigotes after acridine orange (AO) staining. Note the increased AO fluorescence signal in 2-BP parasites (left panel), which corresponds to increased fluorescence in large vacuoles at the posterior cell end (right panel). Bars = 5 μm. (TIF 279 kb)

Additional file 4: **Figure S4.** Effect of incubation with 130 μM palmitate on mitochondrial potential of *Trypanosoma cruzi* epimastigotes. Analysis by flow cytometry using rhodamine-123 shows a decrease of 27.5% in the mitochondrial membrane potential in palmitate-treated cells. 100 μM CCCP: positive control. (TIF 99 kb)
