# Supplementary figures and images for: Treatment of Trypanosoma cruzi with 2-bromopalmitate alters morphology, endocytosis, differentiation and infectivity
Source: BMC Cell Biol. 2018 Aug 31;19:19. doi: 10.1186/s12860-018-0170-3 (PMC6119340; doi:10.1186/s12860-018-0170-3)

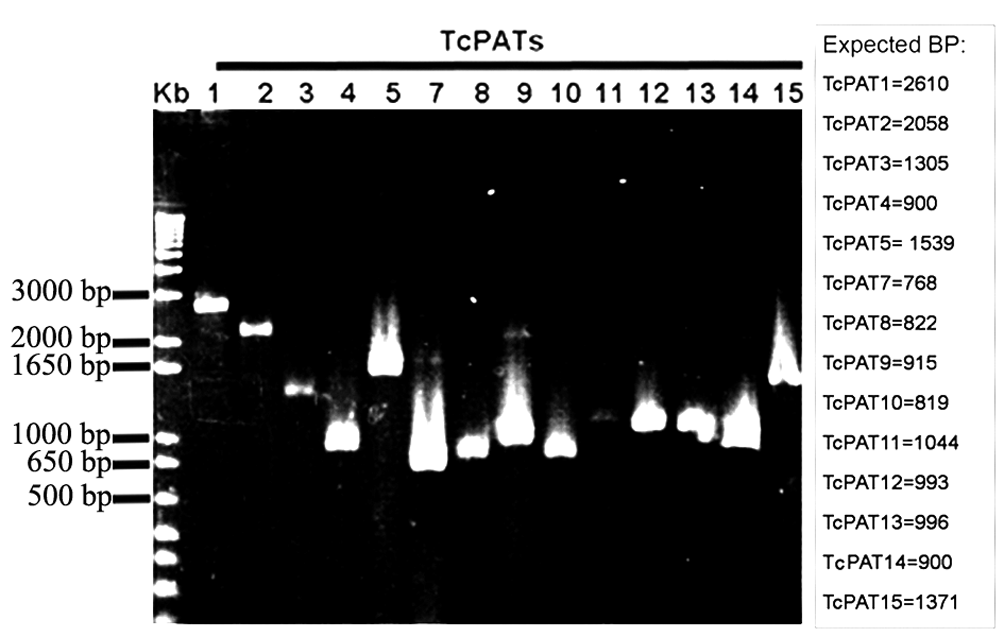

Supplement: Supplementary file 1 — Figure S1. Trypanosoma cruzi PATs genes amplification by PCR as analyzed by 1% agarose gel. Note the expected amplifications for all PATs genes, except TcPAT6. Kb = 1 Kb plus ladder. (TIF 141 kb) [file 12860_2018_170_MOESM1_ESM.tif]

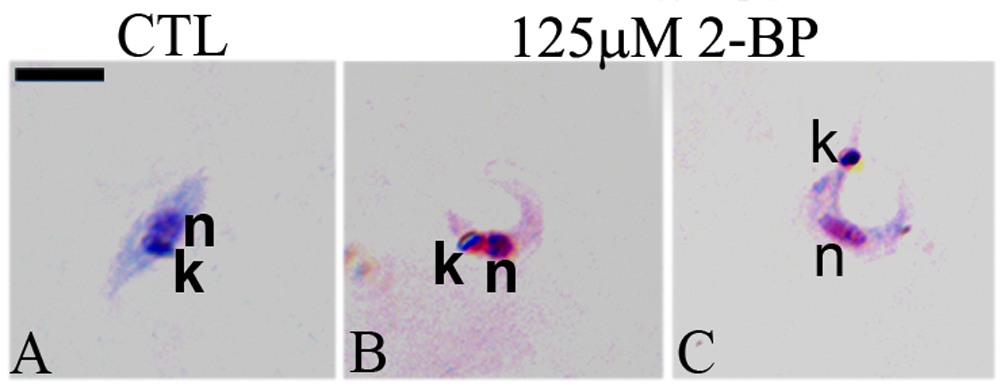

Supplement: Supplementary file 2 — Figure S2. 48 h-old Trypanosoma cruzi intracellular parasites isolated by cavitation after 24 h with 125 μM 2-BP. A) Control isolated amastigote incubated in DMEM medium with 0.125% DMSO; B) Treated parasite with an intermediate trypomastigote-like morphology, with kinetoplast close to the nucleus; C) Treated parasite showing the typical trypomastigote form. n = nucleus; k = kinetoplast. Bars = 5 μm. (TIF 332 kb) [file 12860_2018_170_MOESM2_ESM.tif]

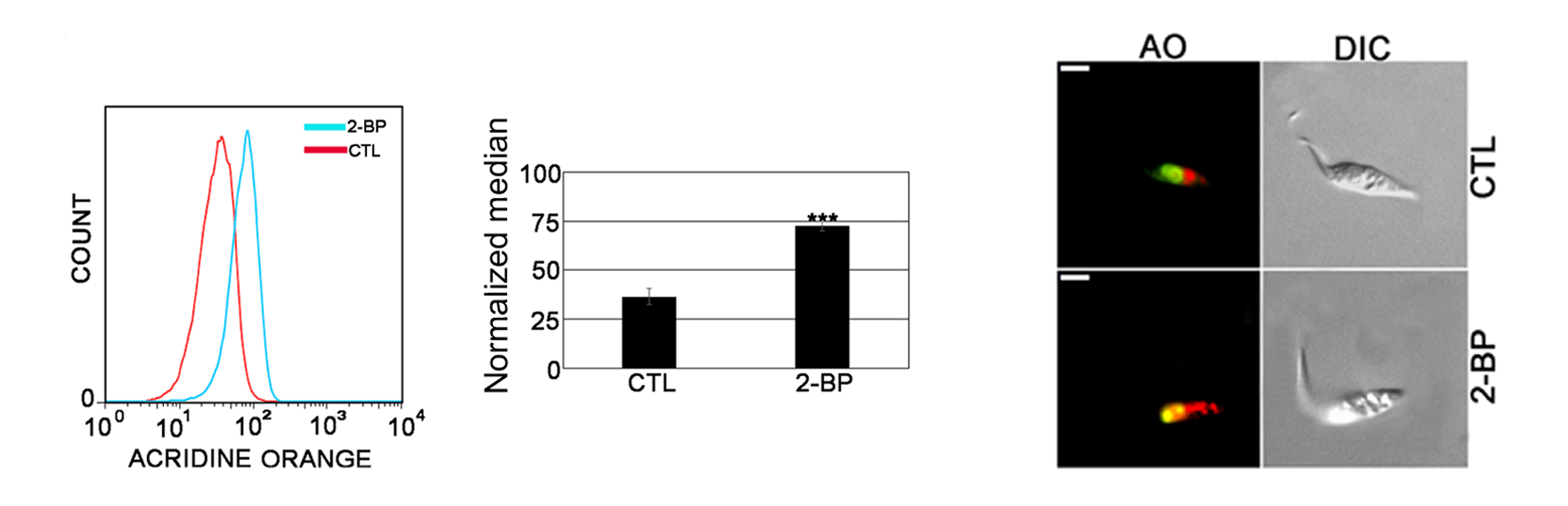

Supplement: Supplementary file 3 — Figure S3. Characterization of acid compartments in control (CTL) and 2-BP-treated epimastigotes after acridine orange (AO) staining. Note the increased AO fluorescence signal in 2-BP parasites (left panel), which corresponds to increased fluorescence in large vacuoles at the posterior cell end (right panel). Bars = 5 μm. (TIF 279 kb) [file 12860_2018_170_MOESM3_ESM.tif]

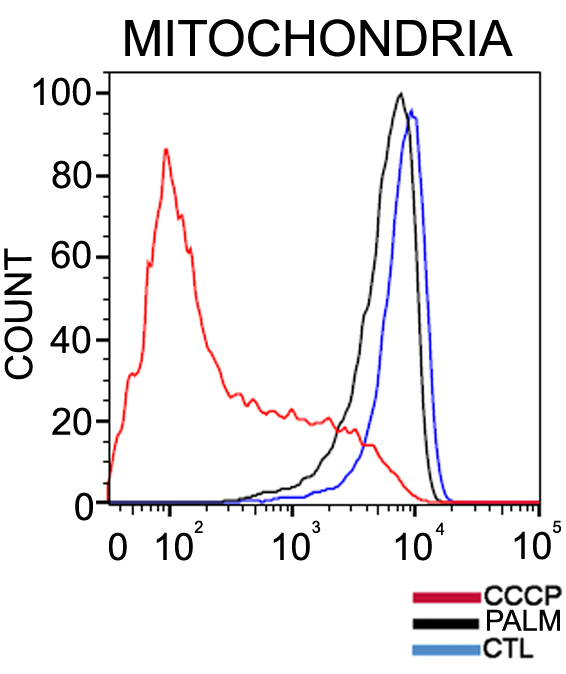

Supplement: Supplementary file 4 — Figure S4. Effect of incubation with 130 μM palmitate on mitochondrial potential of Trypanosoma cruzi epimastigotes. Analysis by flow cytometry using rhodamine-123 shows a decrease of 27.5% in the mitochondrial membrane potential in palmitate-treated cells. 100 μM CCCP: positive control. (TIF 99 kb) [file 12860_2018_170_MOESM4_ESM.tif]
